# Supplementary material for: TcdB of Clostridioides difficile Mediates RAS-Dependent Necrosis in Epithelial Cells
Source: Int J Mol Sci. 2022 Apr 12;23(8):4258. doi: 10.3390/ijms23084258 (PMC9024770; doi:10.3390/ijms23084258)
Supplement: Supplementary file 1 [file ijms-23-04258-s001.zip › ijms-1638295-supplementary.pdf]

TcdB of *Clostridioides difficile* mediates Ras-dependent necrosis in epithelial cells

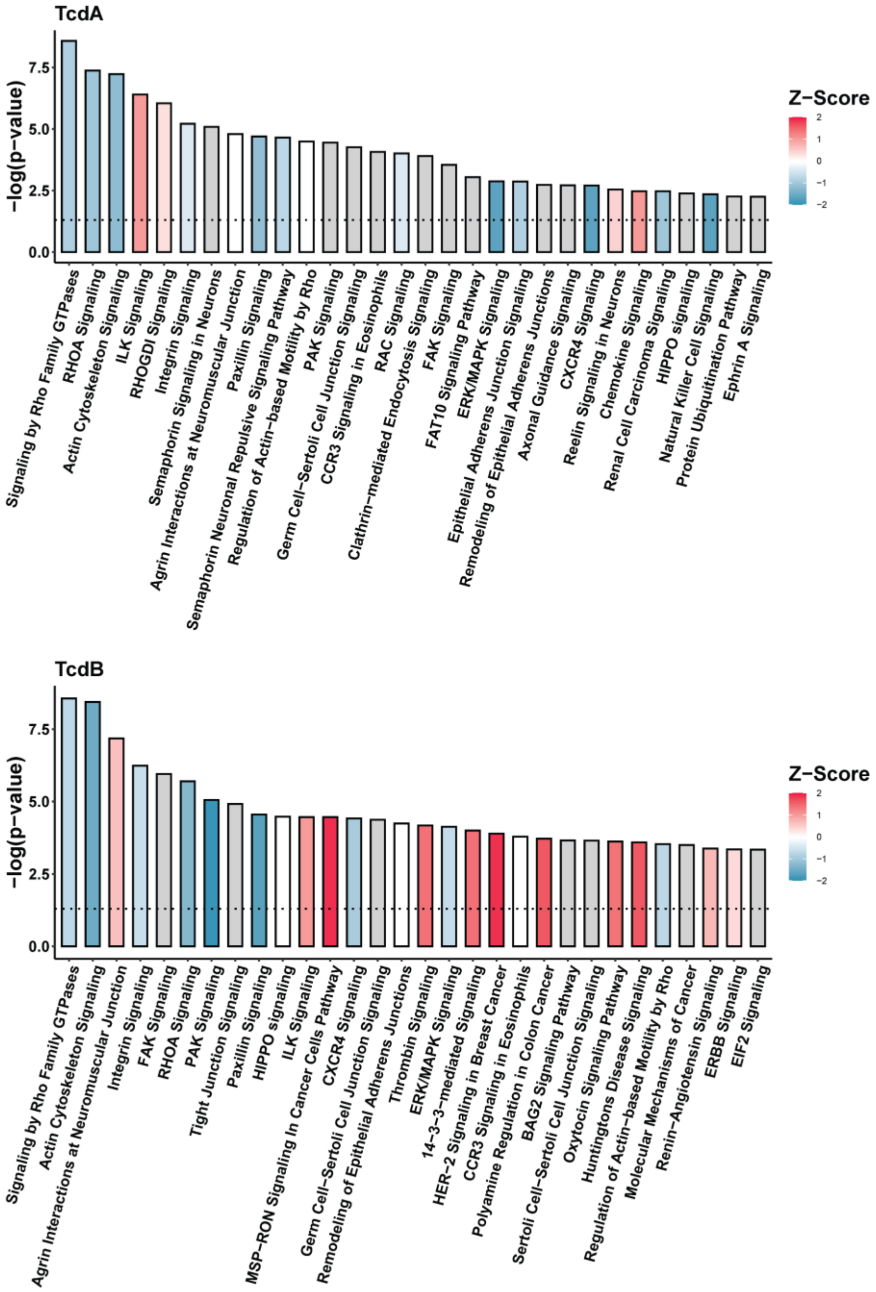

Figure S1: Igenuity Pathway Analysis of HEp-2 cells treated with 20 nM TcdA or 2 nM TcdB for 8h

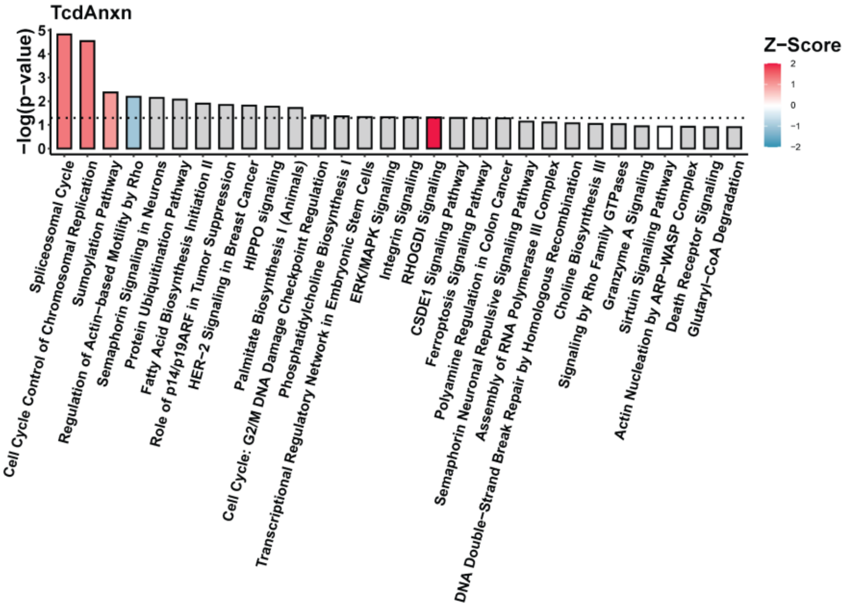

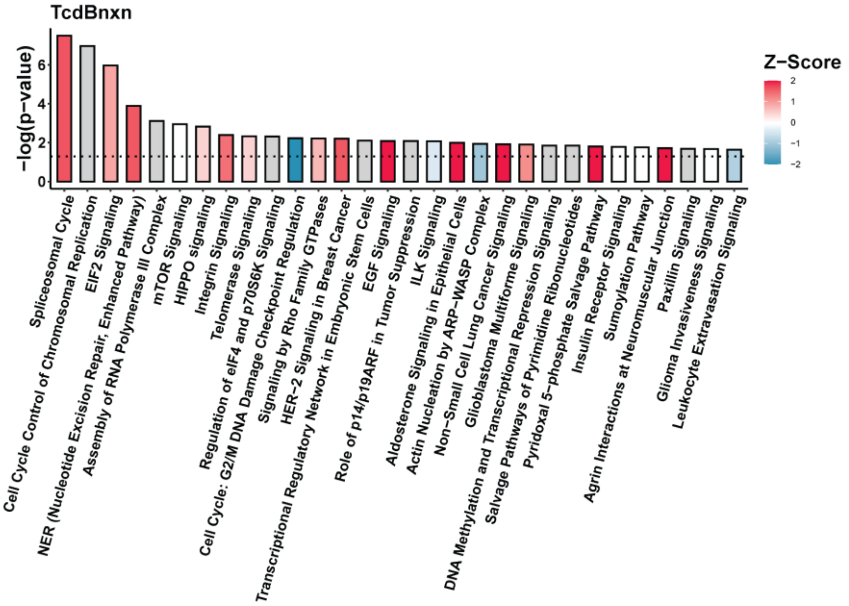

Figure S2: Igenuity Pathway Analysis of HEp-2 cells treated with 20 nM $TcdA_{NXN}$ or 2 nM $TcdB_{NXN}$ for 8h

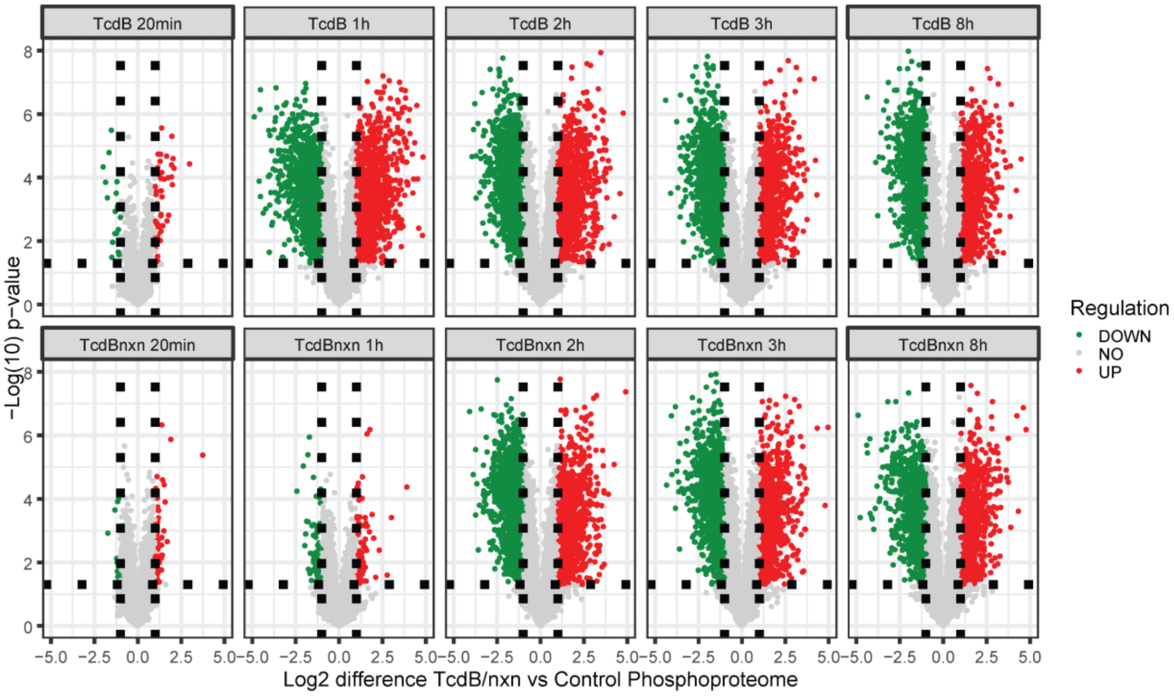

Figure S3: Volcano plot of the Phosphoproteome of HEp-2 cells treated with 2 nM TcdB or 2 nM $TcdB_{NXN}$ at five different time points

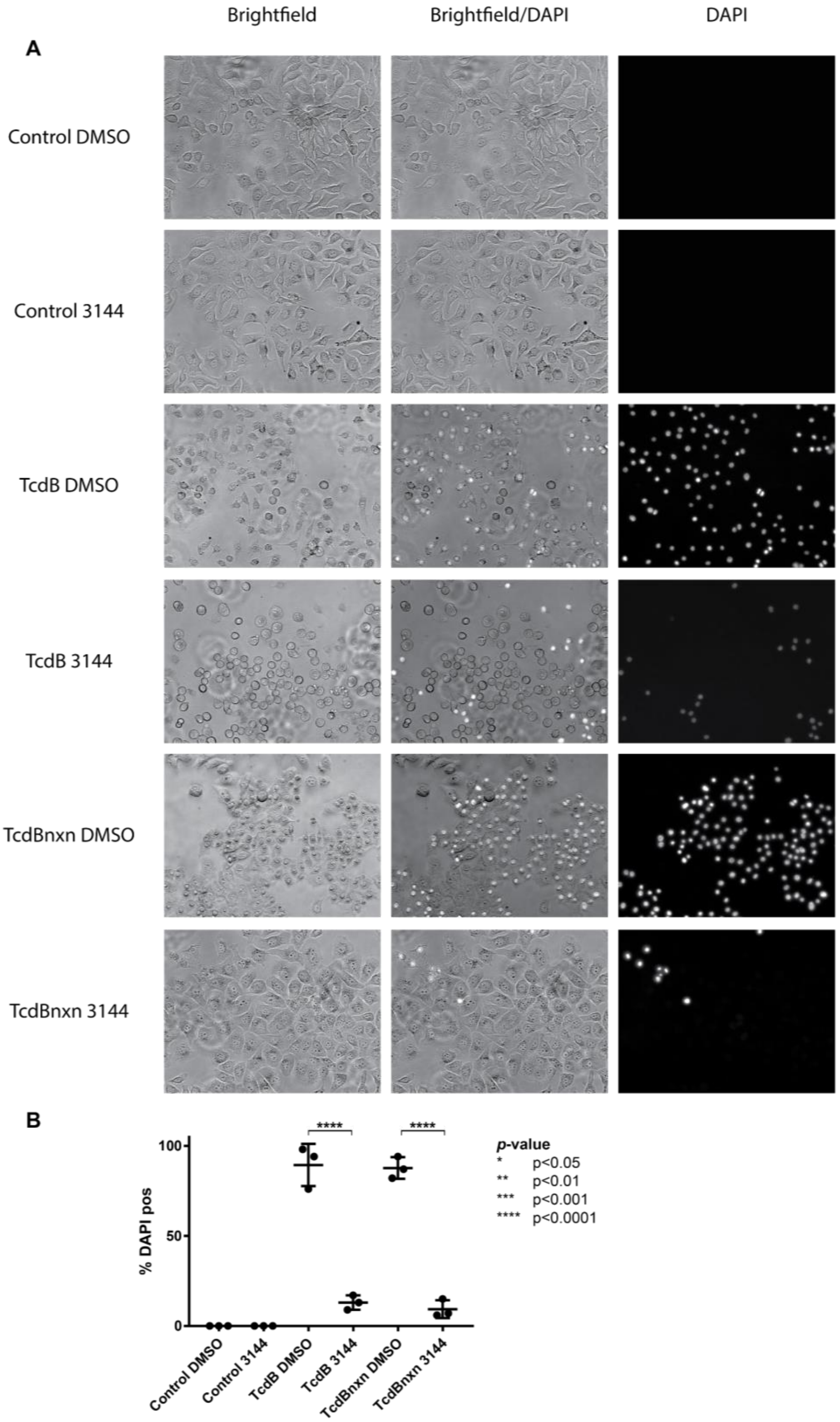

Figure S4: DAPI staining of HEp-2 cells treated with 2 nM TcdB or $TcdB_{NXN}$ for 8h and 17 h pretreatment with 1 µM pan Ras inhibitor 3144 or DMSO. (B) Percentage of DAPI positive cells on total cells. N=3

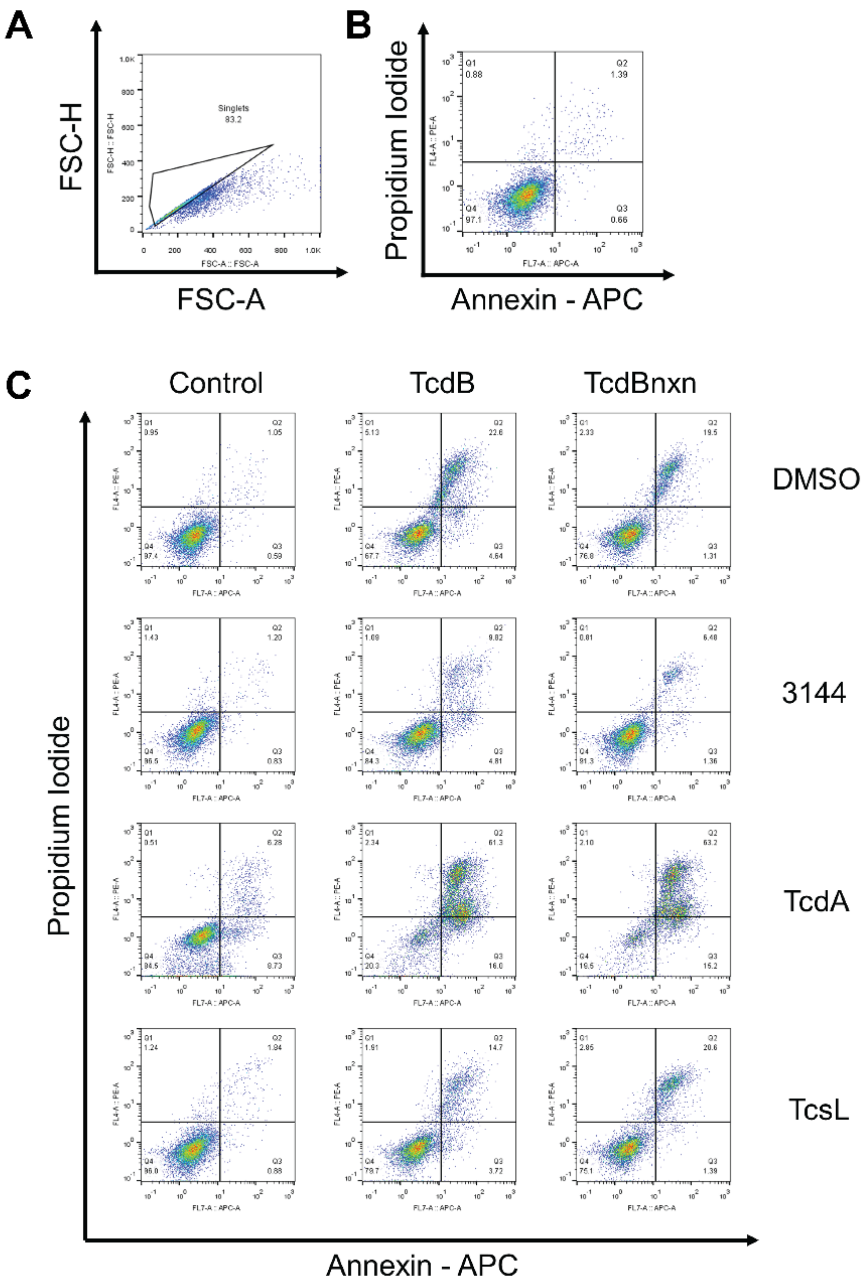

Figure S5: Gating strategy; (A) Gating on single cells and on stained controls (B). (C) Panel shows representative flow cytometry plots of treated HEp-2 cells.

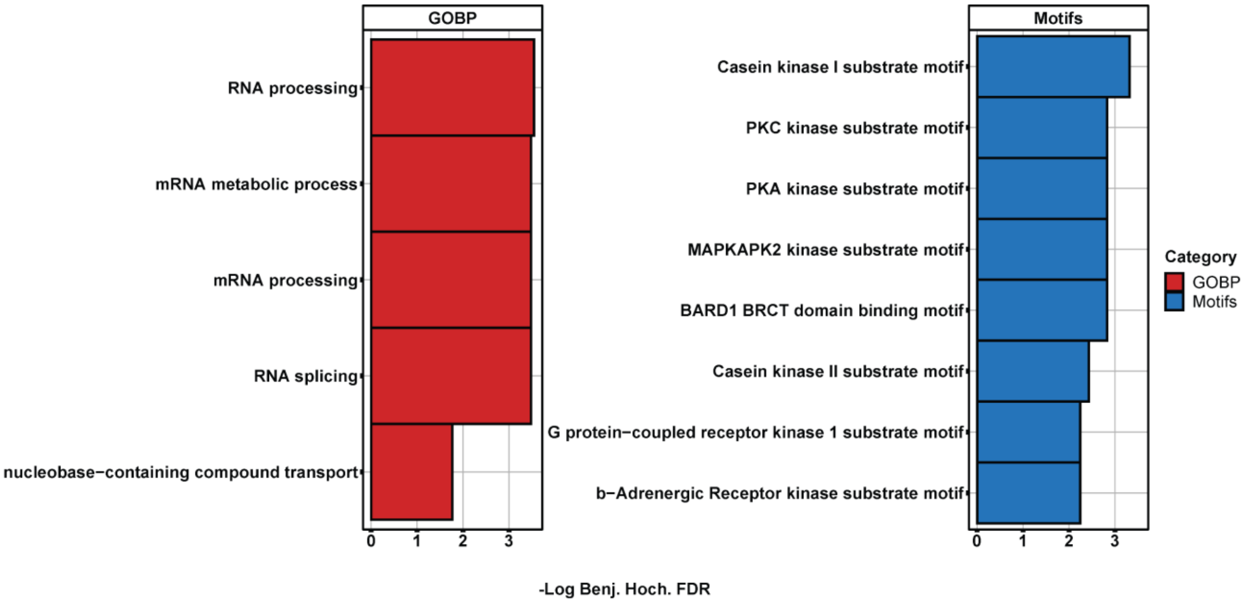

Figure S6: Fisher´s exact test enriched biological GO terms and kinase motifs of row cluster 6, Figure 7A

Table S1: Top 20 up and down regulated phosphosites for 2 nM $TcdB_{NXN}$ vs Control after 8h

Gene names	Protein names	-Log p-value TcdBnxn vs Control	Log2 Difference 8h TcdBnxn vs Ctrl	Phosphorylated amino acid residue
SLC16A1	Monocarboxylate transporter 1	3.44037	4.42294	S-213
ZNF326	DBIRD complex subunit ZNF326	2.71898	4.05955	S-270
SRSF1	Serine/arginine-rich splicing factor 1	3.43242	3.76631	S-238
HTATSF1	HIV Tat-specific factor 1	2.40049	3.55765	S-453
NUCKS1	Nuclear ubiquitous casein and cyclin-dependent kinase substrate 1	3.37798	3.54146	S-61
EIF3C	Eukaryotic translation initiation factor 3 subunit C;Eukaryotic translation initiation factor 3 subunit C-like protein	2.58739	3.4853	S-18;18
CBX3	Chromobox protein homolog 3	2.06419	3.43547	S-93
SRSF5	Serine/arginine-rich splicing factor 5	2.74017	3.33972	S-233
C9orf78	Uncharacterized protein C9orf78	3.32318	3.2572	S-261
TP53BP1	Tumor suppressor p53-binding protein 1	4.66156	3.21471	S-523
NEFH	Neurofilament heavy polypeptide	2.19011	3.18359	S-710
ARPP19	cAMP-regulated phosphoprotein 19	2.36887	3.17081	T-22
ARPP19	cAMP-regulated phosphoprotein 19	2.42874	3.12464	S-23
TRA2B	Transformer-2 protein homolog beta	2.7515	3.05198	S-99
HMGA1	High mobility group protein HMG-I/HMG-Y	2.19072	3.01434	S-102
HMGA1	High mobility group protein HMG-I/HMG-Y	2.19072	3.01434	S-103
SRRM2	Serine/arginine repetitive matrix protein 2	3.43291	3.0016	S-2044
TRIP12	E3 ubiquitin-protein ligase TRIP12	2.57563	2.99058	S-1427
SEC62	Translocation protein SEC62	2.74653	2.95789	T-155
TPD52	Tumor protein D52	2.48619	2.92311	S-176
NPM1	Nucleophosmin	2.51717	2.90646	S-260
CHRAC1	Chromatin accessibility complex protein 1	1.90197	2.8444	S-124
TMEM214	Transmembrane protein 214	1.83268	2.78925	T-97
SCAF1	Splicing factor, arginine/serine-rich 19	2.09354	-1.88546	S-725
AHNAK	Neuroblast differentiation-associated protein AHNAK	2.11152	-1.90569	T-4100
SCAF1	Splicing factor, arginine/serine-rich 19	2.09244	-1.90948	S-724
CD2BP2	CD2 antigen cytoplasmic tail-binding protein 2	2.4233	-1.94258	S-49
SCAF1	Splicing factor, arginine/serine-rich 19	2.19188	-1.96377	S-719
SCAF1	Splicing factor, arginine/serine-rich 19	2.55591	-1.99488	S-738
DENND4C	DENN domain-containing protein 4C	1.3035	-2.011	S-741
SCAF1	Splicing factor, arginine/serine-rich 19	2.52404	-2.03451	S-734
AHNAK	Neuroblast differentiation-associated protein AHNAK	3.1795	-2.03924	T-3716
SYMPK	Symplekin	4.94176	-2.04386	T-1257
AHNAK	Neuroblast differentiation-associated protein AHNAK	3.2654	-2.08276	S-5857
SYNRG	Synergin gamma	2.30006	-2.0842	S-812
STRN3	Striatin-3	4.06861	-2.13314	S-229
AHNAK2	Protein AHNAK2	2.30644	-2.15785	S-497
EPB41L1	Band 4.1-like protein 1	2.90444	-2.20555	S-650
CHAMP1	Chromosome alignment-maintaining phosphoprotein 1	2.15636	-2.24821	S-355
RPS6	40S ribosomal protein S6	3.62298	-2.28153	S-236
SUN2	SUN domain-containing protein 2	2.32387	-2.29767	S-38
HNRNPC	Heterogeneous nuclear ribonucleoproteins C1/C2	2.5489	-3.2367	S-253
HNRNPC	Heterogeneous nuclear ribonucleoproteins C1/C2	2.5489	-3.2367	S-260

Table S2. Top 20 up and down regulated phosphosites for 2 nM TcdB vs Control after 8h.

Gene name	Protein name	-Log p-value 8h TcdB vs Ctrl	log2 Difference 8h TcdB vs Ctrl	Phosphorylated amino acid residue
SRSF1	Serine/arginine-rich splicing factor 1	5.45461	4.33902	S-238

EIF3C	Eukaryotic translation initiation factor 3 subunit C;Eukaryotic translation initiation factor 3 subunit C-like protein	3.19379	4.30244	S-18
SLC16A1	Monocarboxylate transporter 1	2.81879	4.17174	S-213
ANP32A	Acidic leucine-rich nuclear phosphoprotein 32 family member A;Acidic leucine-rich nuclear phosphoprotein 32 family member D	4.25274	3.81983	S-17
SRSF5	Serine/arginine-rich splicing factor 5	3.89101	3.7264	S-233
MAP1S	Microtubule-associated protein 1S;MAP1S heavy chain;MAP1S light chain	4.28766	3.25659	T-638
AMPD2	AMP deaminase 2	4.49077	3.165	S-192
SRSF7	Serine/arginine-rich splicing factor 7	5.27833	3.08017	S-192
SRSF1	Serine/arginine-rich splicing factor 1	3.33629	2.94106	S-242
AMPD2	AMP deaminase 2	3.49974	2.91126	S-190
TOP2A	DNA topoisomerase 2-alpha	2.81556	2.85334	S-1354
TOMM22	Mitochondrial import receptor subunit TOM22 homolog	4.22145	2.78774	S-15
SNRNP27	U4/U6.U5 small nuclear ribonucleoprotein 27 kDa protein	2.57654	2.73753	S-63
ANLN	Actin-binding protein anillin	3.41146	2.73451	S-800
AHNAK	Neuroblast differentiation-associated protein AHNAK	3.10556	2.68415	S-135
SUB1	Activated RNA polymerase II transcriptional coactivator p15	1.33164	2.6833	S-15
SRRM2	Serine/arginine repetitive matrix protein 2	3.2255	2.59898	S-2044
SUPT16H	FACT complex subunit SPT16	3.51712	2.59065	S-982
SRRM2	Serine/arginine repetitive matrix protein 2	3.36575	2.56584	S-1502
MLF2	Myeloid leukemia factor 2	2.21604	2.52208	S-216
AFAP1	Actin filament-associated protein 1	3.48638	-2.35753	S-548
SH3KBP1	SH3 domain-containing kinase-binding protein 1	2.34006	-2.39926	S-230
FNBP1L	Formin-binding protein 1-like	3.36543	-2.41984	S-295
PAK2	Serine/threonine-protein kinase PAK 2;PAK-2p27;PAK-2p34	2.80196	-2.43674	T-143
PKP3	Plakophilin-3	1.48696	-2.46538	S-313
STK10	Serine/threonine-protein kinase 10	2.65173	-2.54819	S-20
PXN	Paxillin	2.25453	-2.55206	S-258
GIT2	ARF GTPase-activating protein GIT2	3.615	-2.80263	S-514
SCRIB	Protein scribble homolog	3.45906	-2.8124	S-1378
STK10	Serine/threonine-protein kinase 10	2.61972	-2.93563	S-450
PAK2	Serine/threonine-protein kinase PAK 2;PAK-2p27;PAK-2p34	3.7506	-2.95499	S-152
STK10	Serine/threonine-protein kinase 10	4.22532	-3.03676	S-417
PLEKHA6	Pleckstrin homology domain-containing family A member 6	2.77206	-3.04972	S-808
CDC42EP4	Cdc42 effector protein 4	2.61624	-3.10738	S-109
STK10	Serine/threonine-protein kinase 10	3.29143	-3.21989	T-459
DOCK5	Dedicator of cytokinesis protein 5	4.93765	-3.40466	S-1766
LRRC16A	Leucine-rich repeat-containing protein 16A	4.22488	-3.45559	S-968
AHNAK	Neuroblast differentiation-associated protein AHNAK	6.10686	-3.50193	S-5448
ARHGEF7	Rho guanine nucleotide exchange factor 7;Rho guanine nucleotide exchange factor 6	2.38101	-3.88559	S-703
ARHGAP29	Rho GTPase-activating protein 29	5.38266	-3.92731	S-1019

Table S3: Top 20 up and down regulated phosphosites for 20 nM TcdA vs Control after 8h

Gene name	Protein name	-Log p-value 8h TcdA vs Control	Log2 Difference 8h TcdA vs Control	Phosphorylated amino acid residue
ANP32A	Acidic leucine-rich nuclear phosphoprotein 32 family member A;Acidic leucine-rich nuclear phosphoprotein 32 family member D	4.30575	3.11625	S-17
RAB7A	Ras-related protein Rab-7a	3.54531	2.81393	S-72
OSBPL11	Oxysterol-binding protein-related protein 11	4.30985	2.65027	S-286
SLC43A3	Solute carrier family 43 member 3	3.09775	2.5926	S-248
SRRM2	Serine/arginine repetitive matrix protein 2	2.17335	2.4364	S-924
IQGAP1	Ras GTPase-activating-like protein IQGAP1	3.98309	2.29623	T-1434
SRRM2	Serine/arginine repetitive matrix protein 2	2.51355	2.28729	S-2044
MKI67	Antigen KI-67	1.97868	2.25193	S-1169
RICTOR	Rapamycin-insensitive companion of mTOR	2.38097	2.21743	T-1103
HSPB1	Heat shock protein beta-1	3.18738	2.20219	S-15
MARCKSL1	MARCKS-related protein	4.75476	2.20215	T-148
AHNAK	Neuroblast differentiation-associated protein AHNAK	3.8375	2.13231	T-3716
C9orf78	Uncharacterized protein C9orf78	1.972	2.11767	S-261
MLF2	Myeloid leukemia factor 2	2.26578	2.09817	S-216

ZC3H13	Zinc finger CCCH domain-containing protein 13	3.80748	2.0793	S-318
NPM1	Nucleophosmin	1.32106	2.0558	S-260
NPM1	Nucleophosmin	1.30698	2.04199	T-237
SARNP	SAP domain-containing ribonucleoprotein	3.4254	1.98862	S-162
SND1	Staphylococcal nuclease domain-containing protein 1	3.50138	1.90786	S-642
CDC42EP1	Cdc42 effector protein 1	3.70513	1.82723	S-101
AFAP1	Actin filament-associated protein 1	3.23569	-2.78581	S-548
PPFIA1	Liprin-alpha-1	5.12425	-2.82461	S-239
SCRIB	Protein scribble homolog	3.12203	-2.9377	S-1378
GIT2	ARF GTPase-activating protein GIT2	3.72766	-2.95884	S-514
PAK2	Serine/threonine-protein kinase PAK 2;PAK-2p27;PAK-2p34	3.28343	-2.96111	S-141
PAK4	Serine/threonine-protein kinase PAK 4	3.37514	-3.03592	S-181
SH3KBP1	SH3 domain-containing kinase-binding protein 1	1.49173	-3.10339	S-230
PKP3	Plakophilin-3	1.41404	-3.22132	S-313
CD2AP	CD2-associated protein	3.42093	-3.30534	S-458
LRRC16A	Leucine-rich repeat-containing protein 16A	5.13105	-3.35139	S-968
PAK2	Serine/threonine-protein kinase PAK 2;PAK-2p27;PAK-2p34	2.14725	-3.45171	T-143
STK10	Serine/threonine-protein kinase 10	4.10317	-3.71662	T-459
PLEKHA6	Pleckstrin homology domain-containing family A member 6	4.18329	-3.77956	S-808
STK10	Serine/threonine-protein kinase 10	2.90492	-3.79441	S-20
STK10	Serine/threonine-protein kinase 10	3.55378	-3.91594	S-450
DOCK5	Dedicator of cytokinesis protein 5	3.65303	-4.26479	S-1766
AHNAK	Neuroblast differentiation-associated protein AHNAK	5.45783	-4.40452	S-5448
PAK2	Serine/threonine-protein kinase PAK 2;PAK-2p27;PAK-2p34	3.28058	-4.58058	S-152
ARHGAP29	Rho GTPase-activating protein 29	2.90565	-5.22925	S-1019
CDC42EP4	Cdc42 effector protein 4	1.6903	-5.25829	S-109

Table S4. Top 20 up and down regulated phosphosites for 20 nM $TcdA_{NXN}$ vs Control after 8h.

Gene names	Protein names	-Log p-value 8h TcdAnxn vs Control	log2 Difference 8h TcdAnxn vs Control	Phosphorylated amino acid residue
ZNF326	DBIRD complex subunit ZNF326	2.86953805	3.78037504	S-270
NPM1	Nucleophosmin	2.23657898	3.76236365	T-234
BANF1	Barrier-to-autointegration factor;Barrier-to-autointegration factor, N-terminally processed	2.12693794	3.41210695	T-2
BANF1	Barrier-to-autointegration factor;Barrier-to-autointegration factor, N-terminally processed	2.12693794	3.41210695	T-3
HTATSF1	HIV Tat-specific factor 1	2.29077345	3.35167593	S-453
NPM1	Nucleophosmin	2.36770717	3.29404179	T-237
HMGA1	High mobility group protein HMG-I/HMG-Y	2.75372505	3.21558833	S-102
HMGA1	High mobility group protein HMG-I/HMG-Y	2.75372505	3.21558833	S-103
C9orf78	Uncharacterized protein C9orf78	3.18171734	3.07830137	S-261
NPM1	Nucleophosmin	2.3243906	2.95547871	S-260
NPM1	Nucleophosmin	2.41111037	2.94749226	T-237
TPD52	Tumor protein D52	2.19479626	2.92785304	S-176
HIST1H1B	Histone H1.5	2.6173044	2.89047629	S-18
YAP1	Transcriptional coactivator YAP1	1.71489368	2.81191738	S-164
ARPP19	cAMP-regulated phosphoprotein 19	2.16086784	2.69078197	T-22
ARPP19	cAMP-regulated phosphoprotein 19	2.25733297	2.62248252	S-23
SEC62	Translocation protein SEC62	2.72557608	2.59517411	T-155
NEFH	Neurofilament heavy polypeptide	1.84359809	2.58296927	S-710
MAP4	Microtubule-associated protein 4	2.24394684	2.56305776	S-787
MAP1B	Microtubule-associated protein 1B;MAP1B heavy chain;MAP1 light chain LC1	1.62925616	2.52621734	S-1917
SCAF1	Splicing factor, arginine/serine-rich 19	2.29587518	-1.48634512	S-738
HNRNPC	Heterogeneous nuclear ribonucleoproteins C1/C2	2.28031475	-1.5123015	S-260
SCAF1	Splicing factor, arginine/serine-rich 19	2.24571041	-1.51281523	S-734
SCAF1	Splicing factor, arginine/serine-rich 19	1.93855796	-1.53190029	S-724
CHAMP1	Chromosome alignment-maintaining phosphoprotein 1	1.95786445	-1.54998096	S-344
SRRM2	Serine/arginine repetitive matrix protein 2	2.11174631	-1.56595009	T-829
UBA1	Ubiquitin-like modifier-activating enzyme 1	1.75132219	-1.56751171	S-13
NOP2	Probable 28S rRNA (cytosine(4447)-C(5))-methyltransferase	1.73925812	-1.57067848	T-195
SUN2	SUN domain-containing protein 2	1.88655956	-1.57388467	S-38
SCAF1	Splicing factor, arginine/serine-rich 19	1.84490688	-1.57961924	S-725

SCAF1	Splicing factor, arginine/serine-rich 19	1.93740013	-1.60105362	S-719
EIF4B	Eukaryotic translation initiation factor 4B	2.10190228	-1.63887708	S-16
RPAP1	RNA polymerase II-associated protein 1	1.36578536	-1.65051205	T-321
CD2BP2	CD2 antigen cytoplasmic tail-binding protein 2	2.18763859	-1.68055912	S-49
KIAA1671	Uncharacterized protein KIAA1671	2.28217057	-1.68574446	S-1695
DENND4C	DENN domain-containing protein 4C	1.56790364	-1.79020865	S-741
CHAMP1	Chromosome alignment-maintaining phosphoprotein 1	2.33580866	-2.16015097	S-355
HNRNPC	Heterogeneous nuclear ribonucleoproteins C1/C2	2.16814968	-2.2711005	S-253
HNRNPC	Heterogeneous nuclear ribonucleoproteins C1/C2	2.16814968	-2.2711005	S-260
PPP1R12A	Protein phosphatase 1 regulatory subunit 12A	2.12250601	-2.29695863	S-422
